# Human Hepatic Cancer Stem Cells (HCSCs) Markers Correlated With Immune Infiltrates Reveal Prognostic Significance of Hepatocellular Carcinoma

**DOI:** 10.3389/fgene.2020.00112

**Published:** 2020-02-28

**Authors:** Xiaopu Sang, Fenfang Wu, Di Wu, Shan Lin, Jingyi Li, Nan Zhao, Xiaoni Chen, Anlong Xu

**Affiliations:** ^1^ School of Life Sciences, Beijing University of Chinese Medicine, Beijing, China; ^2^ Department of Central Laboratory, Shenzhen Hospital, Beijing University of Chinese Medicine, Shenzhen, China

**Keywords:** cancer stem cell, hepatocellular carcinoma, prognostic biomarker, immune infiltrates, hepatocellular carcinoma

## Abstract

**Background:**

Several markers have been reported to be specific for hepatic cancer stem cells (HCSCs), which is usually thought to be highly associated with poor clinical outcomes. Tumor-infiltrating immune cells act as an important factor for oncogenesis. Little is known about the correlation of HCSC markers to prognosis and immune infiltrates.

**Methods:**

Expression of HCSC markers was analyzed through Oncomine database, Gene Expression Profiling Interactive Analysis (GEPIA) and Integrative Molecular Database of Hepatocellular Carcinoma (HCCDB), respectively. The prognostic effect of HCSC markers was evaluated using Kaplan-Meier plotter in association with different tumor stages, risk factors, and gender. The correlation of HCSC markers to tumor-infiltrating immune cells was tested by Tumor Immune Estimation Resource (TIMER). HCSC markers related gene sets were investigated by GEPIA, with their biological functions being analyzed by Cytoscape software.

**Results:**

The expression level of 10 HCSC markers in HCC was higher than that in normal tissues in at least one database. Among them, high expression of *CD24*, *SOX9,* and *SOX12* was positively correlated with poor prognosis (*CD24*: OS *P* = 0.0012, PFS *P* = 7.9E–05**. *SOX9*: OS *P* = 0.012. *SOX12*: OS *P* = 0.0004, PFS *P* = 0.0013, respectively). However, the expression of *CD13*, *CD34* and *ALDH1A1* was associated with prolonged OS and PFS. *SOX12* was significantly upregulated in poor prognosis of HCC patients with different conditions. Besides, total nine HCSC markers were identified to be positively associated with immune infiltration, including *SOX12*. Furthermore, Toll-like receptor signaling pathway was found to be one major pathway of these HCSC markers related gene networks.

**Conclusion:**

Our results suggest that seven upregulated HCSC markers (*CD90*, *EpCAM*, *CD133*, *CD24*, *SOX9, CK19,* and *SOX12*) are related with poor prognosis and immune infiltration in HCC. In addition, we find that high *SOX12* expression remarkably affect prognosis in male HCC patients but not in female. HCC patients under viral infection or alcohol intake with increased *SOX12* expression had poorer prognosis. Therefore, HCSCs markers likely play an important role in tumor related immune infiltration and *SOX12* might be a potential therapeutic target in patients with HCC.

## Introduction

Liver cancer is the second leading cause of worldwide cancer death in men, and sixth in women (Torre et al., 2015; Ferlay et al., 2019), and it accounts approximately 50% of the total number of cancer cases and deaths in China (Torre et al., 2015). The most common liver cancer (~78%) is hepatocellular carcinoma (HCC), the primary malignant neoplasm derived from hepatocytes (Laursen, 2014; Zhu et al., 2016). It has been known that the tumor-infiltrating immune cells play a key role in tumor microenvironment of HCC, such as tumor-associated macrophages (TAMs) (Werb and Coussens, 2002) and tumor-infiltrating lymphocytes (TILs) (Chen and Mellman, 2013). TAMs produce factors that maintain cancer-related inflammation and potentiate tumor progression (Schoppmann et al., 2002), whereas some TILs may control cancer outcome (Gao et al., 2007). So far emerging immunotherapies of immune checkpoint blockade for HCC, like programmed death-1 (PD-1) and cytotoxic T lymphocyte associated antigen 4 (CTLA-4), are still in the start-up stage compared to other tumors. And the objective response rate to the anti-PD-1 and anti-CTLA-4 treatment is relatively low (Johnston and Khakoo, 2019). Due to the immune-suppressive microenvironment of HCC, new checkpoint blockade inhibitors or combining checkpoint blockade inhibitors with other methods may be needed to reinforce the effect (Prieto et al., 2015). Therefore, it is urgent to clarify tumor-immune interactions and identification of novel immune-related therapeutic targets in HCC.

Hepatic cancer stem cells (HCSCs) are small populations of stem-like hepatocarcinoma cells which has capacity to initiate and maintain HCC growth (Wang et al., 2018). Recent advances of HCSCs have enabled the identification of cell surface protein markers, showed their characteristics of oncogenicity, metastasis and therapeutic resistance. CD133 (PROM1) was first proposed to be a specific HCSC marker in 2006 (Suetsugi et al., 2006). After that, others were identified, including CD90 (THY1), epithelial cell adhesion molecules (EpCAM), CD24, CD13 (ANPEP), CD34, sex determining region Y-box 9 (SOX9), ATP-binding cassette, subfamily G, member 2 (ABCG2), CD44, aldehyde dehydrogenase (ALDH), CK19 (KRT19), sex determining region Y-box 12 (SOX12), and CD47 (Ma et al., 2008; Yang et al., 2008; Yamashita et al., 2009; Haraguchi et al., 2010; Lee et al., 2011; Zhang et al., 2013; Fernando et al., 2015; Kawai et al., 2015; Park et al., 2015; Li W. et al., 2017; Richtig et al., 2017; Zou et al., 2017; Rodríguez et al., 2018; Wang et al., 2019). Various HCSC markers correlate with diversified forms of cells. Several studies demonstrated that there were different phenotypes of HCSCs in one single HCC specimen with polymorphic cellular features and tumorigenic potentials (Yamashita et al., 2013; Ho et al., 2019), indicating the complexity of HCSCs. Thus, the characteristics and regulatory mechanisms of HCSCs are not fully elucidated.

A better understanding of immune-related mechanism of HCSCs may help to find novel HCSCs-specific targets for immunotherapy. Unfortunately, this knowledge is limited. Hence, here we comprehensively investigated the expressions of HCSC markers and the correlations with prognosis and immune infiltration of HCC patients based on the online database. Furthermore, we constructed HCSC markers-related gene networks and analyzed the function of the networks using bioinformatics tools. The findings in this report reveal the prognostic role of HCSC makers in HCC, and provide a potential relationship and mechanism between HCSCs and immunity.

## Materials and Methods

### Oncomine Database Analysis

The online database Oncomine (https://www.oncomine.org/resource/login.html) is a bioinformatics analysis tool across 18,000 cancer gene expression microarrays (Rhodes et al., 2007). The expression level of HCSC marker genes in HCC was identified in the Gene Summary view of Oncomine database. The following values: *P*-value of 0.01, fold change of 2, gene rank of top 10%, and data type of mRNA were applied to determine the threshold.

### GEPIA Database Analysis

The online database Gene Expression Profiling Interactive Analysis (GEPIA) (http://gepia.cancer-pku.cn/index.html) is a developed interactive website to analyze the RNA sequencing expression data from the TCGA and GTEx projects (Tang et al., 2017). The expression of HCSC marker genes was confirmed by GEPIA in LIHC dataset. The threshold was determined with the following values: *P*-value of 0.01, fold change of 2, and matched normal data of TCGA normal and GTEx data. GEPIA was also used to generate pathological major stage plot, as well as search for genes that has a similar expression pattern with HCSC markers in liver hepatocellular carcinoma (LIHC).

### HCCDB Database Analysis

The online database Integrative Molecular Database of Hepatocellular Carcinoma (HCCDB) (http://lifeome.net/database/hccdb) curated 15 public HCC expression datasets to serve as a one-stop online resource for exploring gene expression of HCC (Lian et al., 2018). The expression of HCSC marker genes was confirmed by HCCDB.

### Kaplan-Meier Plotter Database Analysis

Kaplan-Meier plotter (liver cancer) is an online platform that can assess the RNA-seq data of 364 liver cancer samples (http://kmplot.com/analysis/index.php?p=service&cancer=liver_rnaseq) (Menyhart et al., 2018). The correlation between expression level of HCSC marker genes and survival in liver cancer was analyzed by Kaplan-Meier plotter. Best cutoff, computed hazard ratio (HR) with 95% confidence intervals and *P* value were selected for the analysis of split patients.

### UALCAN Database Analysis

UALCAN is a comprehensive, user-friendly, and interactive web resource for analyzing TCGA transcriptome and clinical patient data (http://ualcan.path.uab.edu/index.html) (Chandrashekar et al., 2017). UALCAN is designed to provide easy access to publicly available cancer OMICS data (TCGA and MET500). In addition, it enables researchers to study the expression level of genes, not only to compare primary tumor with normal tissue samples, but also to compare across different tumor subgroups as defined by pathological cancer stages, tumor grade, gender, and other clinico-pathologic features.

### TIMER Database Analysis

Tumor Immune Estimation Resource (TIMER) is a computational tool to investigate gene expression characterization of tumor-immune interactions in more than 30 cancer types (https://cistrome.shinyapps.io/timer/) (Li T. et al., 2017). TIMER is a resource for systematical evaluations of the clinical impact of different immune cells in diverse cancer types. In this study, the correlation between the expression level of HCSC marker genes and the abundance of immune infiltrates in LIHC dataset was analyzed.

### Gene Ontology and KEGG Pathway Enrichment Analysis

Gene Ontology (GO) is an internationally-standardized gene functional classification system which offers a dynamic-updated controlled vocabulary and a strictly defined concept to comprehensively describe properties of genes and their products in organisms. The functional genes were annotated by GO database (http://www.geneontology.org/) using hypergeometric test to examine the biological functions and pathways. GO functional enrichment analysis provides GO terms which are significantly enriched in the functional genes comparing to the genome background, showing which are connected to the wanted biological functions.

Pathway-based analysis helps further understand genes biological functions. KEGG is the major public pathway-related database of biological systems that integrates genomic, chemical and systemic functional information. KEGG provides a basic knowledge for linking genomes to life through the process of pathway mapping. Pathway enrichment analysis identifies significantly enriched metabolic pathways or signal transduction pathways in the functional genes comparing with the whole genome background.

In this study, an online biological tool DAVID 6.8 and Clue GO were applied to analyze the molecular and functional characteristics of HCSC markers as well as the related gene expression network.

### Statistical Analysis

The KaplanMeier plots was applied to generate survival curves. Subsequently, the outcomes generated from Oncomine were displayed with *P*-values, fold changes, and ranks. HR and *P* or Cox *P*-values from a log-rank test were used to display the results of KaplanMeier plots, and GEPIA. Furthermore, spearman's correlation and statistical significance were applied to evaluate the correlation of gene expression, and the strength of the correlation was determined using the absolute values. *P*-values < 0.05 were considered statistically significant.

## Results

### Transcriptional Levels of HCSC Markers and Correlation With Pathological Parameters in Patients With HCC

**Figure 1:**
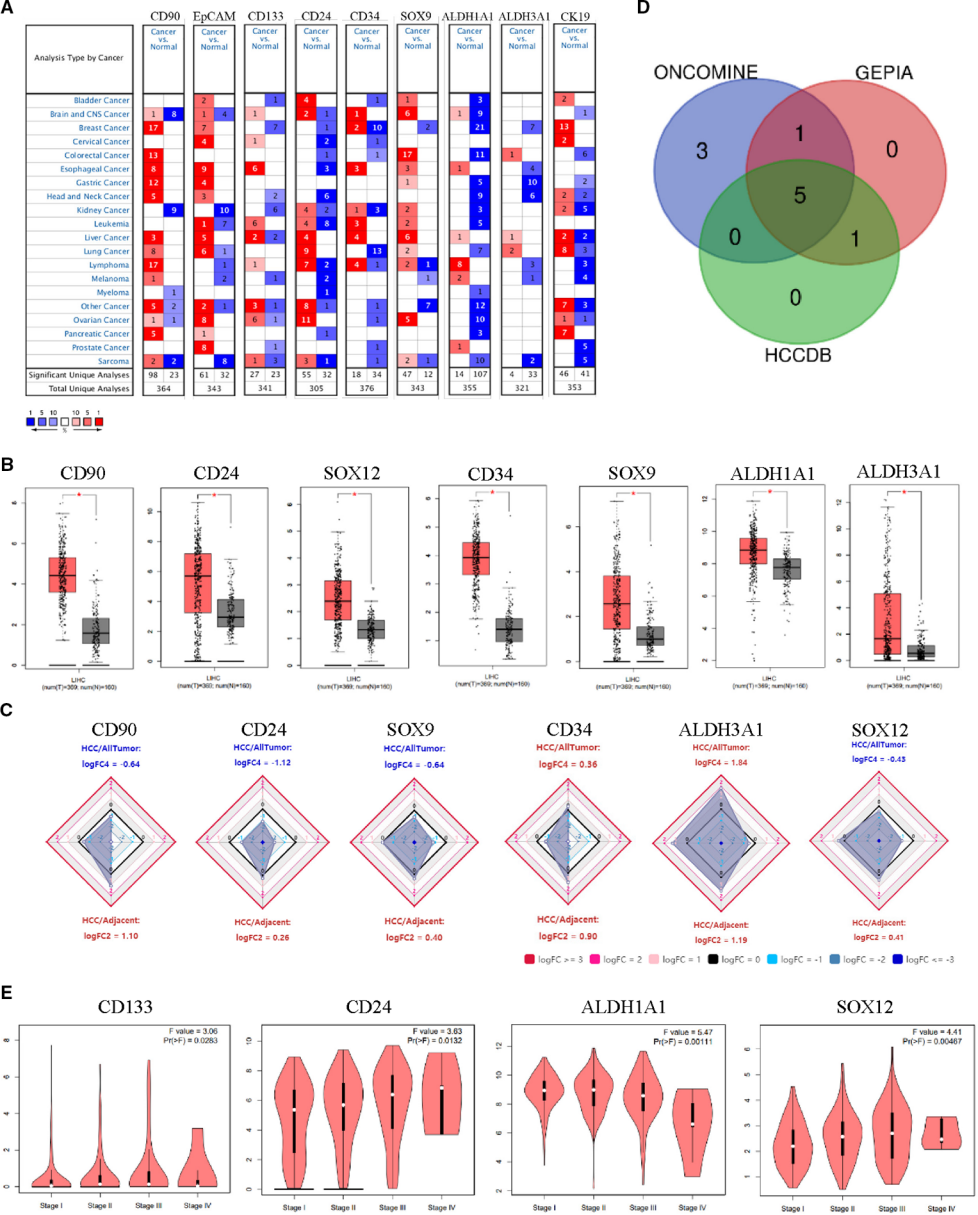
HCSC markers expression levels in HCC. **(A)** Increased HCSC markers in data sets of HCC compared with normal tissues in Oncomine database. Cell color is determined by the best gene rank percentile for the analyses within the cell. **(B)** Increased HCSC markers in data sets of HCC compared with normal tissues in GEPIA. Asterisk: *P* < 0.01. **(C)** Increased HCSC markers in data sets of HCC compared with normal tissues in HCCDB. **(D)** Wayne diagrams of the three database results. **(E)** HCSC markers expression levels at tumor major stages in HCC in GEPIA.

To determine the differences between expression level of HCSC markers in HCC and normal tissues, the mRNA levels of CD90, EpCAM, CD133, CD24, CD13, CD34, SOX9, ABCG2, CD44, ALDH1A1, ALDH3A1, CK19, SOX12, and CD47 in HCC and normal tissues were analyzed based on Oncomine, GEPIA and HCCDB database, respectively. The results from different databases showed to be a little different from each other (**Supplementary Figure 1**). The mRNA expression levels of nine HCSC markers were up-regulated in patients with HCC in Oncomine database, while seven and six were up-regulated in GEPIA database and HCCDB, respectively (**Figures 1A–C**). Among them, *CD90*, *SOX9*, *CD34*, *CD24,* and *ALDH3A1* were significantly increased in all three databases (**Figure 1D**). The *P* value of the five HCSC markers from 12 datasets in HCCDB was listed in **Table 1**.

**Table 1:** P value of the five increased HCSC markers in HCCDB database.

Dataset	Source	CD90	SOX9	CD34	CD24	ALDH3A1
HCCDB1	GSE22058	1.17E–26	2.85E–01	1.96E–38	NA	3.05E–06
HCCDB3	GSE25097	5.21E–18	6.57E–07	5.54E–35	NA	2.60E–07
HCCDB4	GSE36376	1.10E–100	6.80E–31	8.70E–36	7.42E-28	3.39E–15
HCCDB6	GSE14520	5.27E–23	5.67E–17	6.63E–36	NA	1.16E–04
HCCDB7	GSE10143	NA	2.73E–01	3.16E–09	NA	8.65E–09
HCCDB11	GSE46444	7.41E–01	3.75E–01	7.56E–01	1.56E–02	1.82E–02
HCCDB12	GSE54236	6.53E–02	1.38E–01	1.63E–11	8.27E–01	1.03E–04
HCCDB13	GSE63898	8.52E–16	1.29E–01	1.30E–03	5.30E–05	7.35E–25
HCCDB15	TCGA-LIHC	5.74E–21	1.10E–02	5.56E–31	1.32E–03	1.07E–05
HCCDB16	GSE64041	2.71E–06	3.48E–01	3.00E–17	6.12E–01	1.58E–06
HCCDB17	GSE76427	1.73E–05	6.89E–03	5.77E–01	1.03E–01	3.17E–01
HCCDB18	ICGC-LIRI-JP	6.33E–46	2.88E–06	2.70E–53	2.90E–10	3.77E–25

Moreover, the expression of HCSC markers with tumor major stage of HCC were analyzed by GEPIA. *CD133* (*P *= 0.0283), *CD24* (*P *= 0.0132), *SOX12* (*P *= 0.0047), and *ALDH1A1* (*P *= 0.0011) were significantly varied, respectively, whereas other HCSC markers showed no significant difference (**Figure 1E**, **Supplementary Figure 2A**). To further confirm the results, the expression of these varied genes with different tumor stages were analyzed by UALCAN database. The results indicated that the expression level of *CD24* (*P* = 0.0015) and *SOX12* (*P *= 0.0028) was higher in stage III than that in stage I (**Supplementary Figure 2B**). In addition, the expression level of *SOX12* (*P *= 0.0121) was significantly increased on axillary lymph nodes metastasis compared to no regional lymph node metastasis in HCC (**Supplementary Figure 2C**).

We then asked whether the variation of HCSC markers expression was consistent with the gender since a higher incidence of HCC was shown in men than that in women. As a result, the expression of *ABCG2*, *ALDH1A1,* and *ALDH3A1* was significantly increased in male HCC patient compared to female, but *EpCAM*, *CD24*, *CD13,* and *CK19* showed the opposite result (**Table 2**). This indicated that gender might be an important factor to influence HCSC markers.

**Table 2:** Expression of HCSC markers in HCC based on patient's gender in UALCAN database.

Gene name	TPM (median)		*P* value
	Male (n = 245)	Female (n = 117)	
CD90	29.145	32.935	0.0802
EpCAM	0.262	0.693	**0.0023**
CD133	0.016	0.022	0.0521
CD24	29.988	83.881	**0.0066**
CD13	151.728	187.890	**0.0003**
CD34	19.673	18.711	0.7202
SOX9	5.439	5.785	0.3324
ABCG2	4.692	2.574	**2.33E-06**
CD44	6.759	5.179	0.1274
ALDH1A1	701.452	400.749	**1.65E-07**
ALDH3A1	2.733	0.435	**0.0024**
CK19	0.486	0.493	**0.0147**
SOX12	5.003	7.082	0.1456

TPM, Transcript per million. Bolded text in p value means statistically significant.

### Association of HCSC Markers Expression With Prognosis in HCC Patients

**Figure 2:**
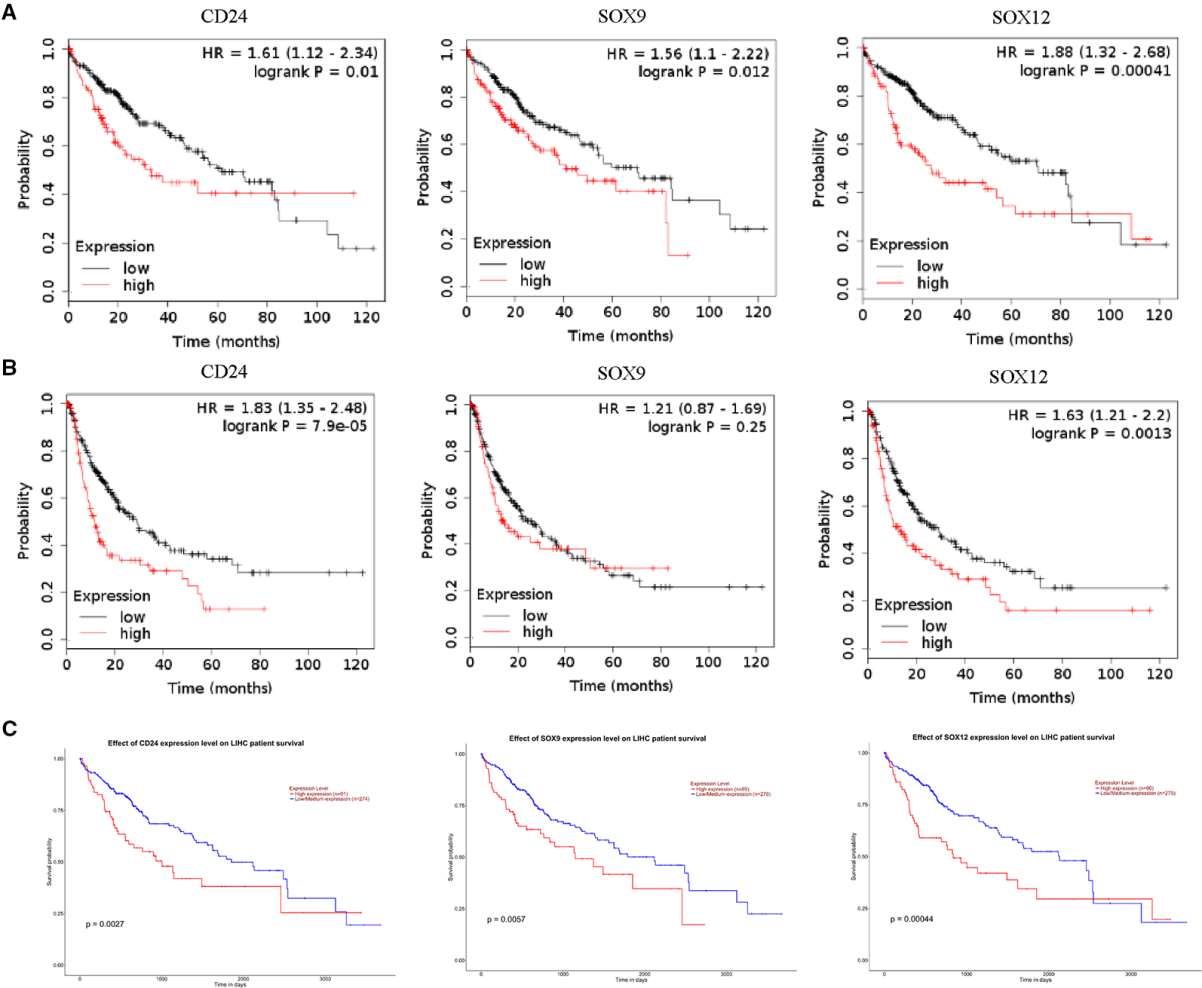
Association of HCSC markers expression levels and prognosis of HCC. **(A)** Correlation of HCSC markers high expression levels with OS of HCC, n = 364, **(B)** correlation of HCSC markers high expression levels with PFS of HCC, n = 370, red font means negative correlation, green font means positive correlation.

Overall survival (OS) is the period from randomization to death in any cases, which is often considered to be the best end-point of efficacy in cancer clinical trials. Progression-free survival (PFS) refers to the period from randomization to tumor progression or death, reflect both tumor growth, and can be evaluated before confirming the survival benefit. To estimate the influence of HCSC markers expression on prognosis of HCC, the correlation of HCSC markers mRNA expression with OS and PFS of HCC were analyzed using Kaplan-Meier Plotter database. The results of all HCSC markers were shown in **Supplementary Figure 3**. Among them, high expression of *CD13*, *CD34* and *ALDH1A1* was negatively correlated with poor prognosis (*CD13*: OS *P* = 0.0012, PFS *P* = 0.0004. *CD34*: OS *P* = 0.0018, PFS *P* = 0.003. *ALDH1A1*: OS *P* = 0.024, PFS *P* = 0.035.). On the contrary, high expression of *CD24*, *SOX9,* and *SOX12* was positively correlated with poor prognosis (*CD24*: OS *P* = 0.0012, PFS *P* = 7.9E-05. *SOX9*: OS *P* = 0.012. *SOX12*: OS *P* = 0.0004, PFS *P* = 0.0013.) (**Figures 2A, B**). We also evaluate the effect of HCSC markers mRNA expression level on HCC patient survival by UALCAN database, and obtain the similar results (**Figure 2C**).

From the above results, we noticed that HCSC markers may have two sides on HCC survival, and each of them had different performance. Hence, the correlation of expression of these markers with OS and PFS of HCC in different tumor stages were further analyzed. In the early stages of HCC, high expression of *EpCAM* (OS *P* = 0.015, PFS *P* = 0.023), *CD133* (OS *P* = 0.028), *CD13* (OS *P* = 0.0044, PFS *P* = 0.0065), *CD34* (OS *P* = 0.017, PFS *P* = 0.0074), and *CK19* (OS *P* = 0.0067, PFS *P* = 0.014) had positive correlation with good prognosis, respectively, while *CD44* (OS *P* = 0.0087), *SOX12* (OS *P* = 0.033), and *CD24* (PFS *P* = 7.9E-05) had negative correlation (**Supplementary Figure 4**). Meanwhile, in stage III and IV of HCC, high expression of *ABCG2* (OS *P* = 0.02), *ALDH1A1* (OS *P* = 0.042), *EpCAM* (*P* = 0.038), *CD133* (PFS *P* = 0.045), *CD13* (PFS *P* = 0.042) *CD44* (PFS *P* = 0.031), and *CD47* (PFS *P* = 0.011) had positive correlation with good prognosis, respectively, while *CD24* (OS HR *P* = 0.011), *SOX9* (OS *P* = 0.0025) and *SOX12* (OS *P* = 0.0005, PFS *P* = 0.0077) had negative correlation (**Supplementary Figure 5**).

Considering the effect of gender on prognosis, the correlation of HCSC markers expression with OS and PFS of HCC were evaluated based on patients' gender. Unexpectedly, the high expression of *SOX12* (Male: OS *P* = 5.9E-5, PFS *P* = 2E-5. Female: OS *P* = 0.3, PFS *P* = 0.42) showed close correlation with poor prognosis of male HCC patient, but not the female (**Supplementary Figures 6A, B**). Interestingly, the expression of *SOX12* in the male and female was similar (**Supplementary Figure 6C**, **Table 2**).

### High Expression of SOX12 Impacts the Prognosis in HCC Patients With Risk Factors

**Figure 3:**
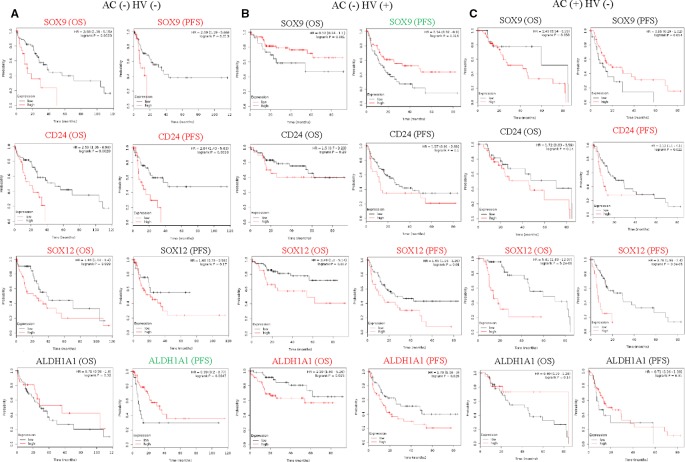
Association of HCSC markers expression levels with prognosis of HCC with risk factors. **(A)** Correlation of HCSC markers high expression levels with OS (n = 91) and PFS (n = 91) of HCC without alcohol consumption (AC) and hepatitis virus (HV), **(B)** correlation of HCSC markers high expression levels with OS (n = 111) and PFS (n = 114) of HCC with HV, **(C)** correlation of HCSC markers high expression levels with OS (n = 76) and PFS (n = 78) of HCC with AC, red font means negative correlation, green font means positive correlation, black font means no correlation.

Alcohol consumption and hepatitis virus are risk factors for HCC (Forner et al., 2018). Unique correlations between HCSC markers and HCC survival rate were found under different risk factors by conducting the analysis in Kaplan-Meier Plotter database as well. *SOX12* was up-regulated in poor OS and PFS with alcohol consumption or hepatitis virus. Besides, *SOX12* (OS *P* = 5.2E-06, PFS *P* = 3.3E-05.) showed more significantly negative correlation with prognosis under alcohol consumption than that under hepatitis virus (**Figure 3C**). *CD24* (OS *P* = 0.0028, PFS *P* = 0.0018) and *SOX9* (OS *P* = 0.0023, PFS *P* = 0.013) both significantly up-regulated in poor OS and PFS in HCC without alcohol consumption and hepatitis virus (**Figure 3A**). *ALDH1A1* (OS *P* = 0.025, PFS *P* = 0.026.) and *ALDH3A1* (OS *P* = 0.0016, PFS *P* = 0.0016.) were specially up-regulated in poor OS and PFS with hepatitis virus (**Figure 3B**).

The above results indicated heterogeneity among these HCSC markers from clinical outcomes. Relationships between different HCSC markers and different tumor stages varied greatly with risk factors of prognosis in HCC. Besides, the effect of same HCSC markers on HCC was different under diverse conditions, suggesting the regulatory function of HCSC markers would be intricate.

### Relationship Between HCSC Markers Expression and Immune Infiltration Level in HCC

**Figure 4:**
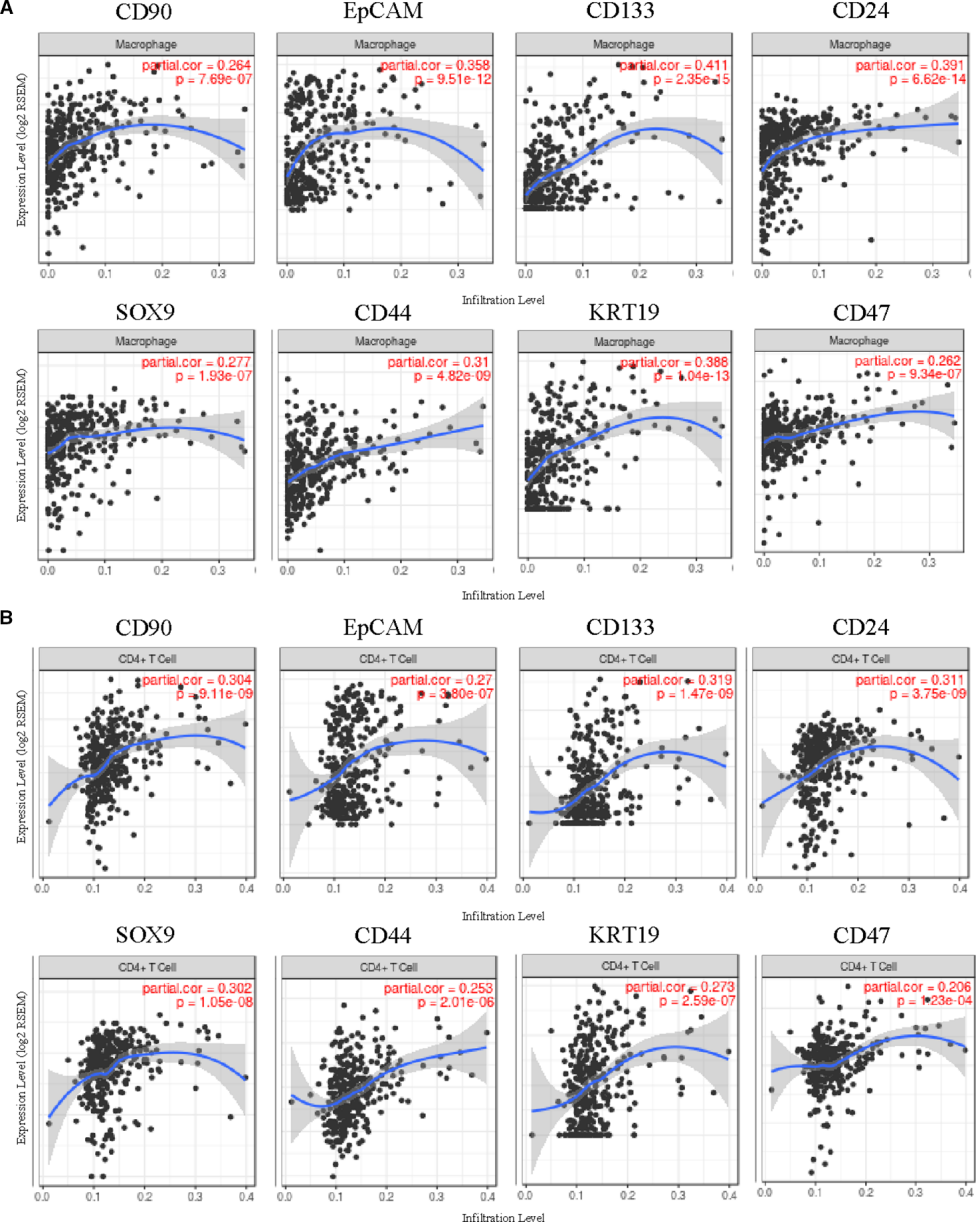
Correlation of seven HCSC markers expression with immune infiltration level in HCC.

Furthermore, the correlation of HCSC markers expressions with immune infiltration level in HCC from TIMER was investigated. The results suggested that some HCSC markers were increased with immune cell infiltration levels in HCC, while others were decreased or had no relationship (**Supplementary Table 1**). Expressions of *CD90*, *EpCAM*, *CD133*, *CD24*, *SOX9*, *CD44*, *CK19,* and *CD47* were positively related to immune infiltration level in HCC, negatively related to tumor purity. Infiltrating levels of macrophage had the most significantly positive correlation with the eight genes, including *CD90* (r = 0.27, *P* = 7.69E-07), *EpCAM* (r = 0.36, *P* = 9.51E-12), *CD133* (r = 0.41, *P* = 2.35E-15), *CD24* (r = 0.39, *P* = 6.62E-14), *SOX9* (r = 0.28, *P* = 1.93E-07), *CD44* (r = 0.31, *P* = 4.82E-09), *CK19* (r = 0.39, *P* = 1.04E-13), and *CD47* (r = 0.26, *P* = 9.34E-07) (**Figure 4A**). Moreover, the second significant correlation was shown with CD4+ T cells, including *CD90* (r = 0.30, *P* = 9.11E-09), *EpCAM* (r = 0.27, *P* = 3.80E-07), *CD133* (r = 0.32, *P* = 1.47E-09), *CD24* (r = 0.31, *P* = 3.75E-09), *SOX9* (r = 0.30, *P* = 1.05E-08), *CD44* (r = 0.25, *P* = 2.01E-06), *CK19* (r = 0.39, *P* = 1.04E-13), and *CD47* (r = 0.21, *P* = 1.23E-04) (**Figure 4B**). In addition, *CD90* and *CD47* also showed remarkable positive correlations with infiltrating levels of dendritic cells (*CD90* r = 0.31, *P* = 5.06E-09, *CD47* r = 0.30, *P* = 1.17E-08), and *CD44* showed remarkable positive correlations with infiltrating levels of dendritic cells (r = 0.36, *P* = 6.16E-12) and neutrophils (r = 0.32, *P* = 1.48E-09) (**Supplementary Table 1**). Besides, *SOX12* was positively related with B cells (r = 0.215, *P* = 6.00E-05), CD8+ cells (r = 0.125, *P* = 2.09E-02) and macrophages (r = 0.208, *P* = 1.09E-04) (**Supplementary Table 1**).

Expressions of *ABCG2*, *ALDH1A1,* and *ALDH3A1* were negatively related with immune infiltration level in HCC, and showed no relation with tumor purity. *CD13* and *CD34* had no significant relationship with immune infiltration level in HCC (**Supplementary Table 1**), and their high expressions were related with good outcomes (**Figure 2**). Those findings suggested that *CD90*, *EpCAM*, *CD133*, *CD24*, *SOX9*, *CD44*, *CK19*, *CD47,* and *SOX12* played specific roles in regulating macrophage infiltration in HCC, which may play an important role in poor prognosis of HCC.

### HCSC Markers Related Gene Regulatory Network in HCC

**Figure 5:**
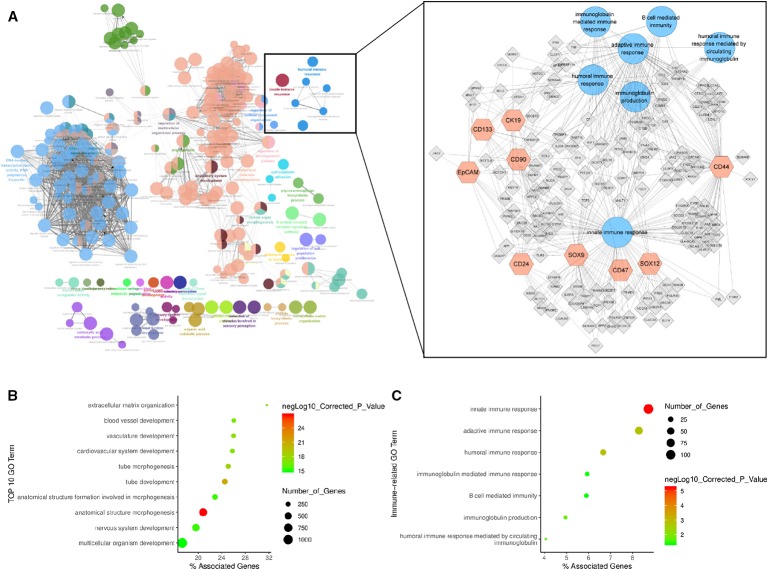
HCSC markers related gene regulatory network. **(A)** HCSC markers related gene regulatory network. The network graph on the left shows all enriched GO items, with each dot representing a GO item. The network graph on the right is a detailed version of the immune-related GO items in the diagram on the left, with each dot representing a GO item, hexagon representing a HCSC mark gene, quadrilateral representing related gene, and line representing a correlation. **(B)** GO enrichment plot of biological function. **(C)** GO enrichment plot of immune related biological function.

To better understand the immune influence of the 9 HCSC markers (*CD90*, *EpCAM*, *CD133*, *CD24*, *SOX9 CD44*, *CK19, CD47,* and *SOX12*) expression in HCC, 4710 positively related genes with similar expression pattern with the nine HCSC markers were detected in HCC dataset of TCGA by GEPIA. Subsequently, the biological functions of the gene set were investigated by ClueGO and CluePedia analysis in Cytoscape software (**Figure 5A**). The majority biological function groups were involved in anatomical structure morphogenesis and development (**Figure 5B**, **Supplementary Table 2**). This was consistent with the properties of stem cells. Besides, GO items of innate immune response, adaptive immune response, humoral immune response, humoral immune response mediated by circulating immunoglobulin, immunoglobulin production and B cell mediated immunity, immunoglobulin mediated immune response were also significantly enriched in the network (**Figure 5C**, **Supplementary Table 2**). And there were 164 genes which took part in these immune GO items. Next, KEGG analysis of the 164 genes and 9 HCSC markers were conducted by DAVID. Hepatitis B and Hepatitis C pathway were significantly enriched, which were very closely related with HCC. Besides, Toll-like receptor signaling pathway, NF-kappa B signaling pathway, RIG-I-like receptor signaling pathway and T cell receptor signaling pathway were also significantly enriched (**Supplementary Figure 7**, **Supplementary Table 3**). These findings suggested that the nine HCSC markers (*CD90*, *EpCAM*, *CD133*, *CD24*, *SOX9*, *CD44*, *CK19*, *SOX12,* and *CD47*) were not only associated with immune infiltration, but also might impact the immune regulation.

## Discussion

Cancer stem cells (CSCs) have been identified in various human cancers (Sukowati, 2019). It was assumed that tumor growth is fueled by small numbers of tumor stem cell hidden in cancer, just as the renewal of healthy tissue (Clevers, 2011; Batlle and Clevers, 2017). Moreover, recent researches demonstrated that the CSCs are bound up with treatment resistance, tumor relapse and metastasis (Jordan et al., 2006). These findings may explain why tumor recurrence is the almost unavoidable outcome after radiation or chemotherapy. An increasing number of studies suggest that CSCs may be more profoundly impact on the cancer prognosis than we thought (Batlle and Clevers, 2017). Therefore, finding therapeutic targets on CSCs could be a more effective way for cancer treatment, including HCC. HCSCs are hierarchical cell populations of HCC, which are able to initiate and maintain tumor growth, and they have the dual properties of normal stem cells and tumor cells (Sukowati, 2019). As far as we know, CD90, EpCAM, CD133, CD24, CD13, CD34, SOX9, ABCG2, CD44, ALDH, CK19, SOX12, and CD47 are widely recognized as HCSC markers (Ma et al., 2008; Yang et al., 2008; Yamashita et al., 2009; Haraguchi et al., 2010; Lee et al., 2011; Zhang et al., 2013; Fernando et al., 2015; Kawai et al., 2015; Park et al., 2015; Li W. et al., 2017; Richtig et al., 2017; Zou et al., 2017; Rodríguez et al., 2018; Wang et al., 2019), whose combination may result in a wide variety of HCSC phenotypes. Up to date, the majority of HCSC studies focus on identification of the markers for the enriched cell populations that have high tumor initiation ability in immune-deficient mice. In the field of clinical research of human subjects, there is almost no report describing prognosis values of different HCSC markers, which may conform the cell line and animal experiment. In addition, few reports focus on the relationship between HCSC markers and immune infiltration in HCC. Here, we for the first time reported the expression level of 14 HCSC markers which correlate to the prognosis of HCC under different conditions. Interestingly, we find that increased SOX12 expression can impact the prognosis of male HCC patients, and patients with viral infection and alcohol intake. Furthermore, our analyses show that in HCC immune infiltration levels are correlated with nine HCSC markers. Thus, our study provides insights in understanding the potential role of HCSC markers in tumor immunology.

In this study, we evaluated the mRNA expression level of the 14 HCSC markers in HCC by ONCOMINE, GEPIA, and HCCDB online database. The mRNA expression level of 10 HCSC markers was up-regulated in HCC in at least one database, including CD90, EpCAM, CD133, CD24, CD34, SOX9, ALDH1A1, ALDH3A1, CK19, and SOX12 (**Figures 1A–C**). Next, the expression of HCSC markers with tumor major stages of HCC was analyzed by GEPIA and UALCAN database. The expression level of *CD24* and *SOX12* in stage III was higher than that in stage I (**Figure 1E**, **Supplementary Figure 2B**), indicating that *CD24* and *SOX12* may have a role in terminal stage of HCC. Besides, high level of *SOX12* was significantly associated with axillary lymph nodes metastasis (**Supplementary Figure 2**). Previous study of immunohistochemistry staining for CD24 on human HCC tissue samples as well as their non-tumor counterparts showed there were 0% to 16% in the HCC specimens, whereas there was no CD24 expression in the non-tumor counterparts (Lee et al., 2011). It has also been proved that mRNA expression of SOX12 was dramatically upregulated in HCC tissues than in adjacent non-tumorous tissues. And mRNA expression of SOX12 was much higher in primary HCC tissues from patients who developed metastasis than that from those without metastasis (Huang et al., 2015).

Next, the influence of HCSC markers expression on prognosis of HCC was analyzed by Kaplan-Meier Plotter database. High expression of *CD24*, *SOX9,* and *SOX12* was negatively correlated with prognosis. In contrary, high expression of *CD13*, *CD34,* and *ALDH1A1* was positively correlated with prognosis (**Figure 2**). We also analyzed the correlation of HCSC markers expression with OS and PFS of HCC in different tumor stages. High expression of *CD24* and *SOX12* were both correlated with poor prognosis in stage I to IV, and *SOX9* was only correlated with poor prognosis in stage III and IV (**Supplementary Figures 4** and **5**). Immunohistochemistry of 166 HCC surgical specimens showed that compared to SOX9^−^ patients, SOX9^+^ patients had significantly poorer recurrence-free survival, stronger venous invasion (Kawai et al., 2016). Our results on CD24, SOX9, and SOX12 are consistent with previous studies (Lee et al., 2011; Huang et al., 2015; Kawai et al., 2016). So far, most studies have not focused on mRNA expression of HCSC markers in different tumor stages. Our results indicated the significant distinction of tumor stages for certain HCSC markers expression. These findings emphasized a noticeable role of CD24, SOX9, and SOX12 in carcinogenesis and tumor progression in HCC. What we didn't expect was that many HCSC markers with high expression are negatively correlated with poor prognosis, such as *EpCAM*, *CD133,* and *CD13* in stage I to IV, *CD34,* and *CK19* in stage I and II, *ABCG2*, *ALDH1A1*, and *CD44* in stage III and IV (**Supplementary Figures 4** and **5**). As we know, liver has the ability of regeneration, and most of these markers are expressed in human liver multipotent progenitor cells (Dan et al., 2006; Kamiya and Inagaki, 2015). This suggests that HCSC markers may have duo functions for carcinogenic and regenerative mechanisms. Single marker may have limited effect on the poor prognosis of HCC. Hence, it is necessary to test HCSC markers in enough amount of cases to reveal the heterogeneity among cancer patients. At the same time, we have to bear in mind that these markers are also related with normal hepatic stem cell, which can facilitate tissue regeneration (Salama et al., 2010; Yoon et al., 2011; Rahman et al., 2014). Besides, in our results, high expression of *CD90*, *EpCAM*, *CD133*, or *CD44* was not significantly correlate with prognosis in HCC, while it was not the same in other papers (Zhao et al., 2016; Hu et al., 2018; Wendum et al., 2018). This indicated the complexity of HCSCs markers and more researches should be performed.

Hepatitis virus is the main risk factor for HCC (El-Serag, 2012). Hepatitis B and C, the carcinogenic viruses, may lead to HCC by inducing chronic inflammation (Read and Douglas, 2014). In our result, high expression of *SOX12*, *ALDH1A1,* and *ALDH3A1* is associated with poor HCC prognosis in the patients with hepatitis virus (**Figure 3B**). ALDH1A1 and ALDH3A1 are isotypes of ALDH gene family. Aldehyde dehydrogenase, which catalyze the oxidation of aldehydes to their corresponding carboxylic acids, play a major role in alcohol metabolism. Nonetheless, the activity of alcohol dehydrogenase in non-alcoholic fatty liver disease can also be increased (Jelski et al., 2018b). And previous studies have demonstrated the strong interactions between hepatitis virus and alcohol (McCartney et al., 2008). Due to the release of these enzymes from damaged liver cells, the ALDH activity was significantly higher in the sera of patients with hepatitis C than that in healthy persons (Jelski et al., 2018a). These evidences are consistent with our observation on the high expression of *ALDH1A1* and *ALDH3A1* in viral-infected liver cancer with poor prognosis. However, the mechanistic relationship between SOX12 and viral-infected liver cancer need to be further explored.

Previous studies have suggested that alcohol can directly initiate and promote liver cancer development and is associated with tumor progression (Chuang et al., 2015). In our study, high expression of *SOX12* was significantly related with poor prognosis of HCC patients who had alcohol consumption (**Figure 3C**). Not only that, we also found that SOX12 showed a close correlation with poor prognosis of male HCC patient, but no of female (**Supplementary Figures 3A, B**). As we know, gender disparities remarkably influence on the incidence and cumulative risk of liver cancer (Forner et al., 2018). Although previous studies have shown that overexpression of SOX12 promotes HCC metastasis and relates to poor prognosis (Huang et al., 2015; Jiang et al., 2017), there have been no reports about the significant difference of SOX12 in prognosis of HCC patients with different genders or alcohol consumption. As men consistently exceeded women in drinking frequencies and quantities (Wilsnack et al., 2000), the relationship among SOX12, gender and alcohol consumption is obscure, which needs to be further studied. In addition, how the virus or alcohol, gender and other risk factors aggravate the progress of liver cancer through SOX12 also needs our attention in the future. In this respect, virus- and alcohol-related interaction may be involved in the potential carcinogenic mechanism of HCSCs. Immunity plays an important role in the development of cancer and is the part of the adverse effects of both virus and alcohol. Thus, another important aspect of this study is that we investigated the correlation of HCSC markers expressions with immune infiltration level in HCC. Expressions of *CD90, EpCAM, CD133, CD24, SOX9, CD44*, *CK19*, *SOX12,* and *CD47* were positively related with immune infiltration level in HCC, especially with macrophages, and secondly with dendritic cells and neutrophils (**Figure 4**). Most of these genes are correlated with poor prognosis of HCC as analyzed before, implying the level of immune infiltration might be associated with HCSC markers' effect on poor clinical outcomes. The intrahepatic chronic inflammation microenvironment is currently perceived as a factor that facilitates the development of HCC and closely related to clinical prognosis (Galun, 2016), since TAMs produce factors that maintain cancer-related inflammation and potentiate tumor progression (Schoppmann et al., 2002). To further explore the mechanism of HCSCs, we constructed a HCSC markers related gene network, and performed GO and KEGG analysis. The pathway of leukocyte transendothelial migration explained infiltration of macrophages in HCC. Therefore, TAMs-related immune interaction could be a potential mechanism for HCSC markers.

In conclusion, our results suggest that seven upregulated HCSC markers (*CD90*, *EpCAM*, *CD133*, *CD24*, *SOX9, CK19,* and *SOX12*) are related with poor prognosis and immune infiltration in HCC. In addition, we find that high *SOX12* expression remarkably effect prognosis in male HCC patients but not in female. And HCC patients under viral infection or alcohol intake with increased *SOX12* expression had poorer prognosis. Therefore, HCSCs markers likely play an important role in tumor related immune infiltration and *SOX12* might be a potential therapeutic target in patients with HCC.

## Data Availability Statement

Publicly available datasets were analyzed in this study. This data can be found here: https://www.oncomine.org/resource/login.html, http://gepia.cancer-pku.cn/index.html, http://lifeome.net/database/hccdb, http://kmplot.com/analysis/index.php?p=service&cancer=liver_rnaseq, http://ualcan.path.uab.edu/index.html, https://cistrome.shinyapps.io/timer/, http://www.geneontology.org/.

## Author Contributions

Study concept and design: AX and FW. Acquisition of data: FW, XS, DW, and JL. Analysis and interpretation of data: AX, FW, XS, and DW. Statistical analysis: FW, XS, NZ, and XC. Drafting of the manuscript: FW, XS, DW, and SL. Critical revision and final approval of the manuscript: AX. Obtained funding: AX. Administrative, technical support: FW. Study supervision: AX and FW.

## Funding

This study was supported by the National Natural Science Foundation of China (81430099 and 31871244 ), and International Science and Technology Cooperation Program of China (2014DFA32950).

## Conflict of Interest

The authors declare that the research was conducted in the absence of any commercial or financial relationships that could be construed as a potential conflict of interest.

## Supplementary Material

Supplementary Figure 1 14 HCSC markers expression levels in HCC. **(A)** HCSC markers in data sets of HCC compared with normal tissues in Oncomine database. Cell color is determined by the best gene rank percentile for the analyses within the cell. **(B)** HCSC markers in data sets of HCC compared with normal tissues in GEPIA. Asterisk: *P* < 0.01. **(C)** HCSC markers in data sets of HCC compared with normal tissues in HCCDB.

Supplementary Figure 2
**(A)** HCSC markers expression levels at tumor major stages in HCC in GEPIA. **(B)** Expression of CD24 in LIHC based on individual cancer stages. *P* value (Normal V.S Stage1) = 4.61E-12, *P* value (Normal V.S Stage2) = 6.32E-08, *P* value (Normal V.S Stage3) = 3.87E-09, *P* value (Stage1 V.S Stage3) = 0.0015. Expression of SOX12 in LIHC based on individual cancer stages. *P* value (Normal V.S Stage1) = 1.62E-12, *P* value (Normal V.S Stage2) = 3.99E-11, *P* value (Normal V.S Stage3) = 1.28E-09, *P* value (Normal V.S Stage4) = 0.0061, *P* value (Stage1 V.S Stage2) = 0.0427, *P* value (Stage1 V.S Stage3) = 0.0028. **(C)** Expression of CD24 in LIHC based on nodal metastasis status. *P* value (Normal V.S N0) = <1E-12, *P* value (Normal V.S N1) = 0.0064, *P* value (N0 V.S N1) = 0.0121. Expression of SOX12 in LIHC based on nodal metastasis status. *P* value (Normal V.S N0) = 1.62E-12, *P* value (Normal V.S N1) = 0.0433, *P* value (N0 V.S N1) = 0.0121. N0, no regional lymph node metastasis. N1, metastases in 1 to 3 axillary lymph nodes. TPM, Transcript per million.

Supplementary Figure 3 Association of 14 HCSC markers expression levels and prognosis of HCC. **(A)** Correlation of HCSC markers high expression levels with OS of HCC, n=364, **(B)** correlation of HCSC markers high expression levels with PFS of HCC, n=370, red font means negative correlation, green font means positive correlation, black font means no correlation.

Supplementary Figure 4 Association of HCSC markers expression levels and prognosis of HCC in stage I and II. **(A)** Correlation of HCSC markers high expression levels with OS of HCC in stage I and II, n=253, **(B)** correlation of HCSC markers high expression levels with PFS of HCC in stage I and II, n=256, red font means negative correlation, green font means positive correlation, black font means no correlation.

Supplementary Figure 5 Association of HCSC markers expression levels and prognosis of HCC in stage III and IV. **(A)** Correlation of HCSC markers high expression levels with OS of HCC in stage III and IV, n=87, **(B)** correlation of HCSC markers high expression levels with PFS of HCC in stage III and IV, n=90, red font means negative correlation, green font means positive correlation, black font means no correlation.

Supplementary Figure 6 Association of HCSC markers expression levels with prognosis of HCC with different gender. **(A)** Correlation of HCSC markers high expression levels with OS (n=246) and PFS (n=246) of male patient with HCC, **(B)** correlation of HCSC markers high expression levels with OS (n=118) and PFS (n=120) of female patient with HCC, red font means negative correlation, black font means no correlation. **(C)** Expression of HCSC markers in LIHC based on patient's gender. The median TPM and *P* value list in **Table 2**.

Supplementary Figure 7 KEGG enrichment plot of KEGG pathway by functional annotation clustering.

## References

[B1] Batlle E. Clevers H. (2017). Cancer stem cells revisited. Nat. Med. 23 (10), 1124–1134. 10.1038/nm.4409 28985214

[B2] Chandrashekar D. S. Bashel B. Balasubramanya S. Creighton C. J. Ponce-Rodriguez I. Chakravarthi B. (2017). UALCAN: a portal for facilitating tumor subgroup gene expression and survival analyses. Neoplasia 19 (8), 649–658. 10.1016/j.neo.2017.05.002 28732212 PMC5516091

[B3] Chen D. S. Mellman I. (2013). Oncology meets immunology: the cancer-immunity cycle. Immunity 39 (1), 1–10. 10.1016/j.immuni.2013.07.012 23890059

[B4] Chuang S. Lee Y. A. Wu G. Straif K. Hashibe M. (2015). O Alcohol consumption and liver cancer risk: a meta-analysis. Cancer Cause Control 26 (9), 1205–1231. 10.1007/s10552-015-0615-3 26134046

[B5] Clevers H. (2011). The cancer stem cell: premises, promises and challenges. Nat. Med. 17 (3), 313–319. 10.1038/nm.2304 21386835

[B6] Dan Y. Y. Riehle K. J. Lazaro C. Teoh N. Haque J. Campbell J. S. (2006). Isolation of multipotent progenitor cells from human fetal liver capable of differentiating into liver and mesenchymal lineages. Proc. Natl. Acad. Sci. U. S. A. 103 (26), 9912–9917. 10.1073/pnas.0603824103 16782807 PMC1502553

[B7] El-Serag H. B. (2012). Epidemiology of viral hepatitis and hepatocellular carcinoma. Gastroenterology 142 (6), 1264–1273. 10.1053/j.gastro.2011.12.061 22537432 PMC3338949

[B8] Ferlay J. Colombet M. Soerjomataram I. Mathers C. Parkin D. M. Pineros M. (2019). Estimating the global cancer incidence and mortality in 2018: GLOBOCAN sources and methods. Int. J. Cancer 144 (8), 1941–1953. 10.1002/ijc.31937 30350310

[B9] Fernando J. Malfettone A. Cepeda E. B. Vilarrasa-Blasi R. Bertran E. Raimondi G. (2015). A mesenchymal-like phenotype and expression of CD44 predict lack of apoptotic response to sorafenib in liver tumor cells. Int. J. Cancer 136 (4), E161–E172. 10.1002/ijc.29097 25053293

[B10] Forner A. Reig M. Bruix J. (2018). Hepatocellular carcinoma. Lancet 391 (10127), 1301–1314. 10.1016/S0140-6736(18)30010-2 29307467

[B11] Galun E. (2016). Liver inflammation and cancer: the role of tissue microenvironment in generating the tumor-promoting niche (TPN) in the development of hepatocellular carcinoma. Hepatology 63 (2), 354–356. 10.1002/hep.28344 26566854

[B12] Gao Q. Qiu S. Fan J. Zhou J. Wang X. Xiao Y. (2007). Intratumoral balance of regulatory and cytotoxic T cells is associated with prognosis of hepatocellular carcinoma after resection. J. Clin. Oncol. 25 (18), 2586–2593. 10.1200/JCO.2006.09.4565 17577038

[B13] Haraguchi N. Ishii H. Mimori K. Tanaka F. Ohkuma M. Kim H. M. (2010). CD13 is a therapeutic target in human liver cancer stem cells. J. Clin. Invest. 120 (9), 3326–3339. 10.1172/JCI42550 20697159 PMC2929722

[B14] Ho D. W. Tsui Y. Sze K. M. Chan L. Cheung T. Lee E. (2019). Single-cell transcriptomics reveals the landscape of intra-tumoral heterogeneity and stemness-related subpopulations in liver cancer. Cancer Lett. 459, 176–185. 10.1016/j.canlet.2019.06.002 31195060

[B15] Hu Y. Li X. Gao J. (2018). Correlation among the expression of CD133, CD90, and poor survival outcome in hepatocellular carcinoma: a meta-analysis. Int. J. Clin. Exp. Med. 11 (4), 2965–2977.

[B16] Huang W. Chen Z. Shang X. Tian D. Wang D. Wu K. (2015). Sox12, a direct target of FoxQ1, promotes hepatocellular carcinoma metastasis through up-regulating Twist1 and FGFBP1. Hepatology 61 (6), 1920–1933. 10.1002/hep.27756 25704764

[B17] Jelski W. Wolszczak-Biedrzycka B. Zasimowicz-Majewska E. Orywal K. Lapinski T. W. Szmitkowski M. (2018b). Alcohol Dehydrogenase Isoenzymes and Aldehyde Dehydrogenase activity in the serum of patients with non-alcoholic fatty liver disease. Anticancer Res. 38 (7), 4005–4009. 10.21873/anticanres.12688 29970524

[B18] Jelski W. Strumnik A. Orywal K. Lapinski T. W. Swiderska M. Szmitkowski M. (2018a). Activity of alcohol dehydrogenase isoenzymes and aldehyde dehydrogenase in sera of patients with hepatitis C. Arch. Med. Sci. 14 (2), 281–287. 10.5114/aoms.2016.60406 29593800 PMC5868663

[B19] Jiang T. Guan L. Y. Ye Y. S. Liu H. Y. Li R. (2017). MiR-874 inhibits metastasis and epithelial-mesenchymal transition in hepatocellular carcinoma by targeting SOX12. Am. J. Cancer Res. 7 (6), 1310–1321. 28670493 PMC5489780

[B20] Johnston M. P. Khakoo S. I. (2019). Immunotherapy for hepatocellular carcinoma: current and future. World J. Gastroenterol. 25 (24), 2977–2989. 10.3748/wjg.v25.i24.2977 31293335 PMC6603808

[B21] Jordan C. T. Guzman M. L. Noble M. (2006). Cancer stem cells. N. Engl. J. Med. 355 (12), 1253–1261. 10.1056/NEJMra061808 16990388

[B22] Kamiya A. Inagaki Y. (2015). Stem and progenitor cell systems in liver development and regeneration. Hepatol. Res. 45 (1), 29–37. 10.1111/hepr.12349 24773763

[B23] Kawai T. Yasuchika K. Ishii T. Katayama H. Yoshitoshi E. Y. Ogiso S. (2015). Keratin 19, a cancer stem cell marker in human hepatocellular carcinoma. Clin. Cancer Res. 21 (13), 3081–3091. 10.1158/1078-0432.CCR-14-1936 25820415

[B24] Kawai T. Yasuchika K. Ishii T. Miyauchi Y. Kojima H. Yamaoka R. (2016). SOX9 is a novel cancer stem cell marker surrogated by osteopontin in human hepatocellular carcinoma. Sci. Rep-UK 6 (1). 10.1038/srep30489 PMC4960550 27457505

[B25] Laursen L. (2014). A preventable cancer. Nature 516 (7529), S2–S3. 10.1038/516S2a 25470197

[B26] Lee T. K. Castilho A. Cheung V. C. Tang K. H. Ma S. Ng I. O. (2011). CD24(+) liver tumor-initiating cells drive self-renewal and tumor initiation through STAT3-mediated NANOG regulation. Cell Stem Cell 9 (1), 50–63. 10.1016/j.stem.2011.06.005 21726833

[B27] Li W. Ma H. Zhang J. Zhu L. Wang C. Yang Y. (2017). Unraveling the roles of CD44/CD24 and ALDH1 as cancer stem cell markers in tumorigenesis and metastasis. Sci. Rep. 7 (1), 13856. 29062075 10.1038/s41598-017-14364-2 PMC5653849

[B28] Li T. Fan J. Wang B. Traugh N. Chen Q. Liu J. S. (2017). TIMER: a web server for comprehensive analysis of tumor-infiltrating immune cells. Cancer Res. 77 (21), e108–e110. 10.1158/0008-5472.CAN-17-0307 29092952 PMC6042652

[B29] Lian Q. Wang S. Zhang G. Wang D. Luo G. Tang J. (2018). HCCDB: a database of hepatocellular carcinoma expression atlas. Genom. Proteomics Bioinf. 16 (4), 269–275. 10.1016/j.gpb.2018.07.003 PMC6205074 30266410

[B30] Ma S. Chan K. W. Lee T. K. Tang K. H. Wo J. Y. Zheng B. J. (2008). Aldehyde dehydrogenase discriminates the CD133 liver cancer stem cell populations. Mol. Cancer Res. 6 (7), 1146–1153. 10.1158/1541-7786.MCR-08-0035 18644979

[B31] McCartney E. M. Semendric L. Helbig K. J. Hinze S. Jones B. Weinman S. A. (2008). Alcohol metabolism increases the replication of hepatitis C virus and attenuates the antiviral action of interferon. J. Infect. Dis. 198 (12), 1766–1775. 10.1086/593216 18956976

[B32] Menyhart O. Nagy A. Gyorffy B. (2018). Determining consistent prognostic biomarkers of overall survival and vascular invasion in hepatocellular carcinoma. R. Soc. Open Sci. 5 (12), 181006. 10.1098/rsos.181006 30662724 PMC6304123

[B33] Park S. C. Nguyen N. T. Eun J. R. Zhang Y. Jung Y. J. Tschudy-Seney B. (2015). Identification of cancer stem cell subpopulations of CD34(+) PLC/PRF/5 that result in three types of human liver carcinomas. Stem Cells Dev. 24 (8), 1008–1021. 10.1089/scd.2014.0405 25519836 PMC4390116

[B34] Prieto J. Melero I. Sangro B. (2015). Immunological landscape and immunotherapy of hepatocellular carcinoma. Nat. Rev. Gastroenterol. Hepatol. 12 (12), 681–700. 10.1038/nrgastro.2015.173 26484443

[B35] Rahman M. M. Subramani J. Ghosh M. Denninger J. K. Takeda K. Fong G. H. (2014). CD13 promotes mesenchymal stem cell-mediated regeneration of ischemic muscle. Front. Physiol. 4, 402. 10.3389/fphys.2013.00402 24409152 PMC3885827

[B36] Read S. A. Douglas M. W. (2014). Virus induced inflammation and cancer development. Cancer Lett. 345 (2), 174–181. 10.1016/j.canlet.2013.07.030 23941825

[B37] Rhodes D. R. Kalyana-Sundaram S. Mahavisno V. Varambally R. Yu J. Briggs B. B. (2007). Oncomine 3.0: genes, pathways, and networks in a collection of 18,000 cancer gene expression profiles. Neoplasia 9 (2), 166–180. 10.1593/neo.07112 17356713 PMC1813932

[B38] Richtig G. Aigelsreiter A. Schwarzenbacher D. Ress A. L. Adiprasito J. B. Stiegelbauer V. (2017). SOX9 is a proliferation and stem cell factor in hepatocellular carcinoma and possess widespread prognostic significance in different cancer types. PloS One 12 (11), e187814. 10.1371/journal.pone.0187814 PMC5679634 29121666

[B39] Rodríguez M. M. Fiore E. Bayo J. Atorrasagasti C. García M. Onorato A. (2018). 4Mu decreases CD47 expression on hepatic cancer stem cells and primes a potent antitumor T cell response induced by Interleukin-12. Mol. Ther. 26 (12), 2738–2750. 10.1016/j.ymthe.2018.09.012 30301668 PMC6277513

[B40] Salama H. Zekri A. R. Bahnassy A. A. Medhat E. Halim H. A. Ahmed O. S. (2010). Autologous CD34+ and CD133+ stem cells transplantation in patients with end stage liver disease. World J. Gastroenterol. 16 (42), 5297–5305. 10.3748/wjg.v16.i42.5297 21072892 PMC2980678

[B41] Schoppmann S. F. Alitalo K. Jakesz R. Kerjaschki D. Birner P. (2002). Tumor associated macrophages express lymphatic endothelial growth factors and are related to peritumoral lymphangiogenesis. Int. J. Mol. Med. 10 (Supplement 1), S86. 10.1016/S0002-9440(10)64255-1 PMC1867252 12213723

[B42] Suetsugi A. Nagaki M. Aoki H. Motohashi T. Kunisada T. Moriwaki H. (2006). Characterization of CD133(+) hepatocellular carcinoma cells as cancer stem/progenitor cells. Biochem. Bioph. Res. Co. 351 (4), 820–824. 10.1016/j.bbrc.2006.10.128 17097610

[B43] Sukowati C. (2019). Heterogeneity of hepatic cancer stem cells. Adv. Exp. Med. Biol. 1139, 59–81. 10.1007/978-3-030-14366-4_4 31134495

[B44] Tang Z. Li C. Kang B. Gao G. Li C. Zhang Z. (2017). GEPIA: a web server for cancer and normal gene expression profiling and interactive analyses. Nucleic Acids Res. 45 (W1), W98–W102. 10.1093/nar/gkx247 28407145 PMC5570223

[B45] Torre L. A. Bray F. Siegel R. L. Ferlay J. Lortet-Tieulent J. Jemal A. (2015). Global cancer statistics, 2012. CA: A Cancer J. Clin. 65 (2), 87–108. 10.3322/caac.21262 25651787

[B46] Wang N. Wang S. Li M. Y. Hu B. G. Liu L. P. Yang S. L. (2018). Cancer stem cells in hepatocellular carcinoma: an overview and promising therapeutic strategies. Ther. Adv. Med. Oncol. 10, 433535969. 10.1177/1758835918816287 PMC6304707 30622654

[B47] Wang Y. Wu G. Fu X. Xu S. Wang T. Zhang Q. (2019). Aquaporin 3 maintains the stemness of CD133+ hepatocellular carcinoma cells by activating STAT3. Cell Death Dis. 10 (6), 465. 10.1038/s41419-019-1712-0 31197130 PMC6565673

[B48] Wendum D. Layese R. Ganne-Carrie N. Bourcier V. Merabtene F. Cagnot C. (2018). Influence of progenitor-derived regeneration markers on Hepatitis C Virus-Related Cirrhosis Outcome (ANRS CO12 CirVir Cohort). Hepatology 68 (4), 1534–1548. 10.1002/hep.29927 29637581

[B49] Werb Z. Coussens L. M. (2002). Inflammation and cancer. Nature 420 (6917), 860–867. 10.1038/nature01322 12490959 PMC2803035

[B50] Wilsnack R. W. Vogeltanz N. D. Wilsnack S. C. Harris T. R. Ahlstrom S. Bondy S. (2000). Gender differences in alcohol consumption and adverse drinking consequences: cross-cultural patterns. Addiction 95 (2), 251–265. 10.1046/j.1360-0443.2000.95225112.x 10723854

[B51] Yamashita T. Ji J. Budhu A. Forgues M. Yang W. Wang H. Y. (2009). EpCAM-positive hepatocellular carcinoma cells are tumor-initiating cells with stem/progenitor cell features. Gastroenterology 136 (3), 1012–1024. 10.1053/j.gastro.2008.12.004 19150350 PMC2828822

[B52] Yamashita T. Honda M. Nakamoto Y. Baba M. Nio K. Hara Y. (2013). Discrete nature of EpCAM+ and CD90+ cancer stem cells in human hepatocellular carcinoma. Hepatology 57 (4), 1484–1497. 10.1002/hep.26168 23174907 PMC7180389

[B53] Yang Z. F. Ho D. W. Ng M. N. Lau C. K. Yu W. C. Ngai P. (2008). Significance of CD90+ cancer stem cells in human liver cancer. Cancer Cell 13 (2), 153–166. 10.1016/j.ccr.2008.01.013 18242515

[B54] Yoon S. M. Gerasimidou D. Kuwahara R. Hytiroglou P. Yoo J. E. Park Y. N. (2011). Epithelial cell adhesion molecule (EpCAM) marks hepatocytes newly derived from stem/progenitor cells in humans. Hepatology 53 (3), 964–973. 10.1002/hep.24122 21319194

[B55] Zhang G. Wang Z. Luo W. Jiao H. Wu J. Jiang C. (2013). Expression of potential cancer stem cell marker ABCG2 is associated with malignant behaviors of hepatocellular carcinoma. Gastroenterol. Res. Pract. 2013, 782581. 10.1155/2013/782581 24194752 PMC3806359

[B56] Zhao Q. Zhou H. Liu Q. Cao Y. Wang G. Hu A. (2016). Prognostic value of the expression of cancer stem cell-related markers CD133 and CD44 in hepatocellular carcinoma: from patients to patient-derived tumor xenograft models. Oncotarget 7 (30), 47431–47443. 10.18632/oncotarget.10164 27329727 PMC5216952

[B57] Zhu R. X. Seto W. Lai C. Yuen M. (2016). Epidemiology of hepatocellular carcinoma in the Asia-Pacific region. Gut. Liver 10 (3). 10.5009/gnl15257 PMC4849684 27114433

[B58] Zou S. Wang C. Liu J. Wang Q. Zhang D. Zhu S. (2017). Sox12 is a cancer stem-like cell marker in hepatocellular carcinoma. Mol. Cells 40 (11), 847–854. 10.14348/molcells.2017.0129 29127951 PMC5712514

