# Supplementary figures and images for: Human Hepatic Cancer Stem Cells (HCSCs) Markers Correlated With Immune Infiltrates Reveal Prognostic Significance of Hepatocellular Carcinoma
Source: Front Genet. 2020 Feb 28;11:112. doi: 10.3389/fgene.2020.00112 (PMC7058667; doi:10.3389/fgene.2020.00112)

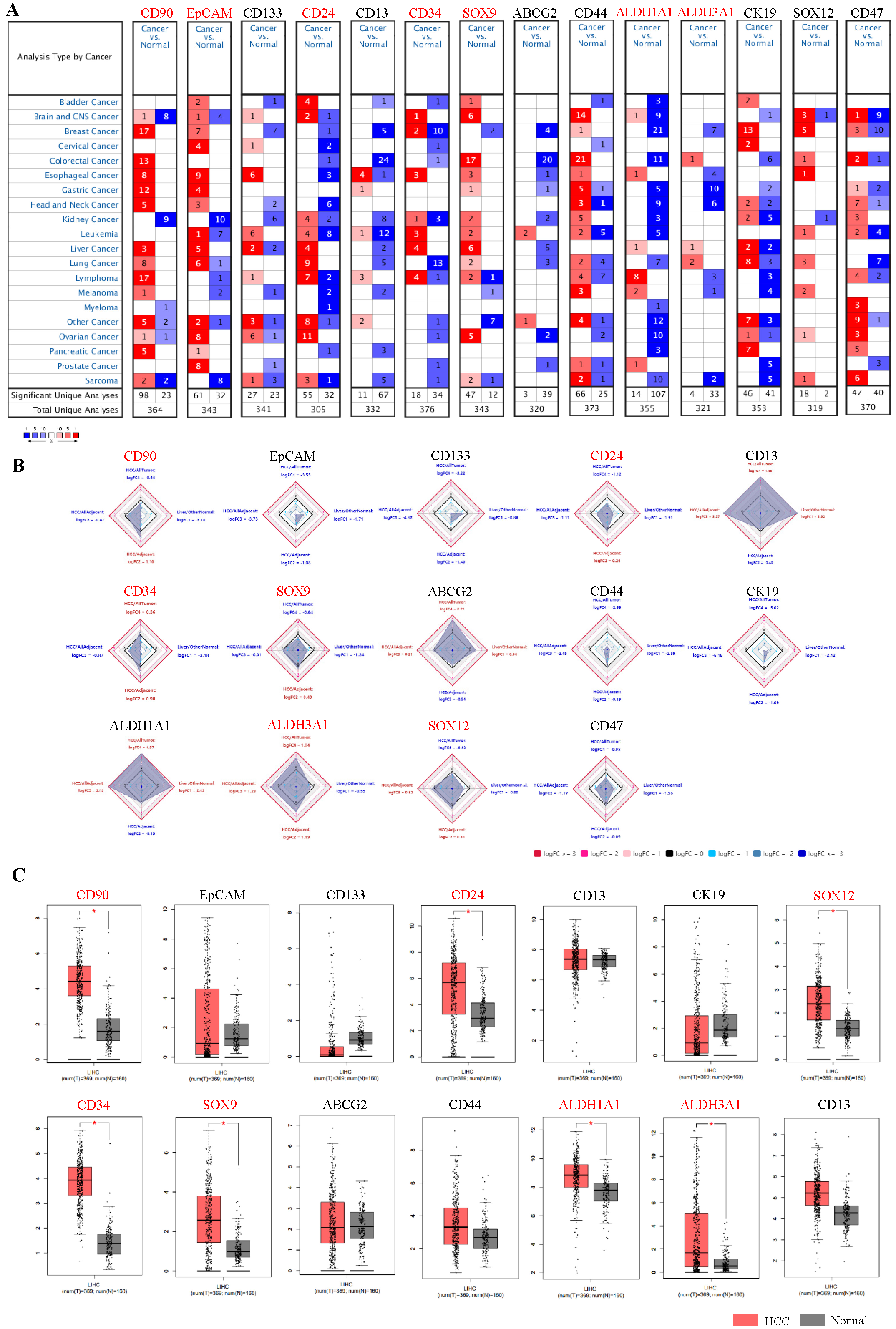

Supplement: Supplementary Figure 1 — 14 HCSC markers expression levels in HCC. (A) HCSC markers in data sets of HCC compared with normal tissues in Oncomine database. Cell color is determined by the best gene rank percentile for the analyses within the cell. (B) HCSC markers in data sets of HCC compared with normal tissues in GEPIA. Asterisk: P < 0.01. (C) HCSC markers in data sets of HCC compared with normal tissues in HCCDB. [file Image_1.tif]

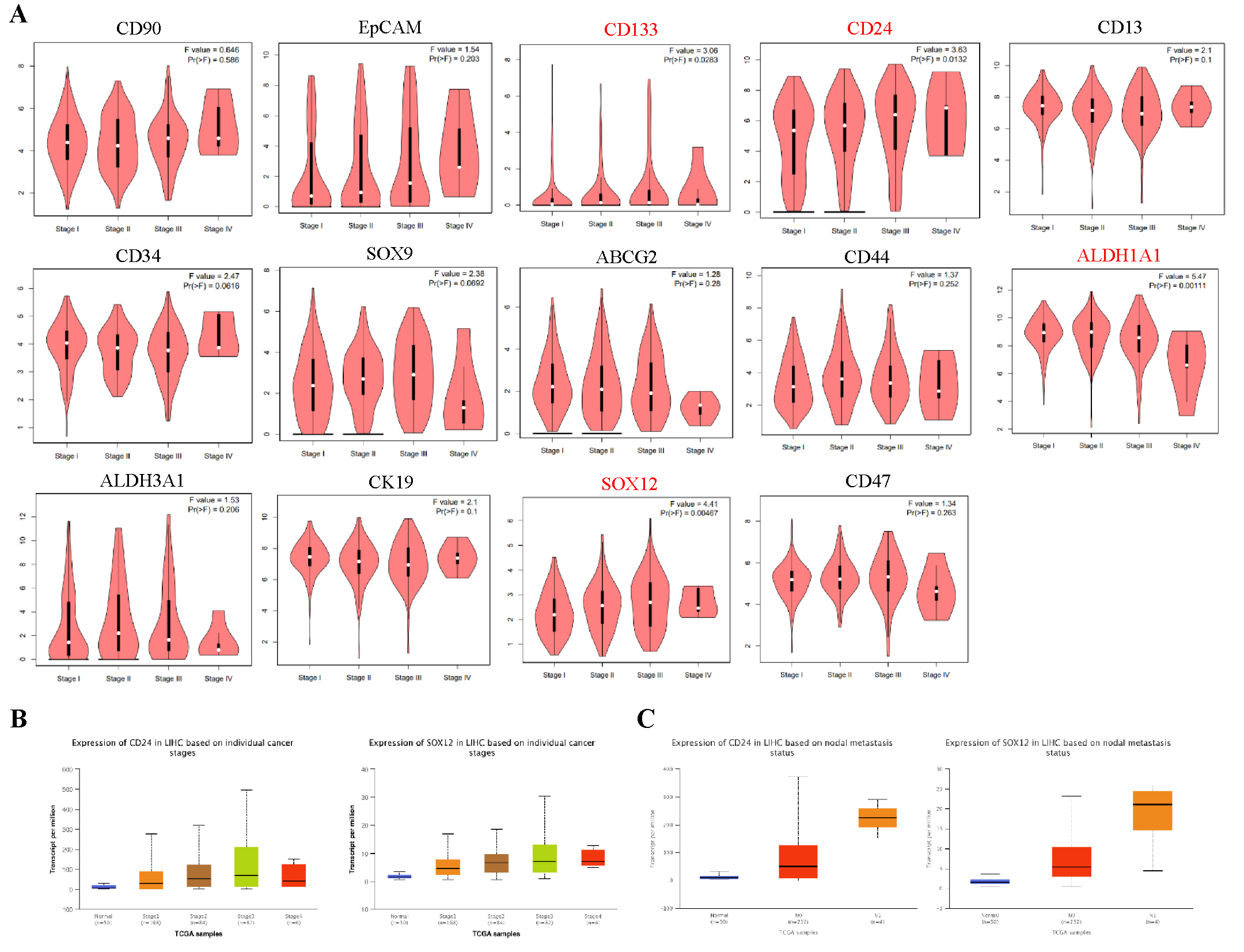

Supplement: Supplementary Figure 2 — (A) HCSC markers expression levels at tumor major stages in HCC in GEPIA. (B) Expression of CD24 in LIHC based on individual cancer stages. P value (Normal V.S Stage1) = 4.61E-12, P value (Normal V.S Stage2) = 6.32E-08, P value (Normal V.S Stage3) = 3.87E-09, P value (Stage1 V.S Stage3) = 0.0015. Expression of SOX12 in LIHC based on individual cancer stages. P value (Normal V.S Stage1) = 1.62E-12, P value (Normal V.S Stage2) = 3.99E-11, P value (Normal V.S Stage3) = 1.28E-09, P value (Normal V.S Stage4) = 0.0061, P value (Stage1 V.S Stage2) = 0.0427, P value (Stage1 V.S Stage3) = 0.0028. (C) Expression of CD24 in LIHC based on nodal metastasis status. P value (Normal V.S N0) = <1E-12, P value (Normal V.S N1) = 0.0064, P value (N0 V.S N1) = 0.0121. Expression of SOX12 in LIHC based on nodal metastasis status. P value (Normal V.S N0) = 1.62E-12, P value (Normal V.S N1) = 0.0433, P value (N0 V.S N1) = 0.0121. N0, no regional lymph node metastasis. N1, metastases in 1 to 3 axillary lymph nodes. TPM, Transcript per million. [file Image_2.tif]

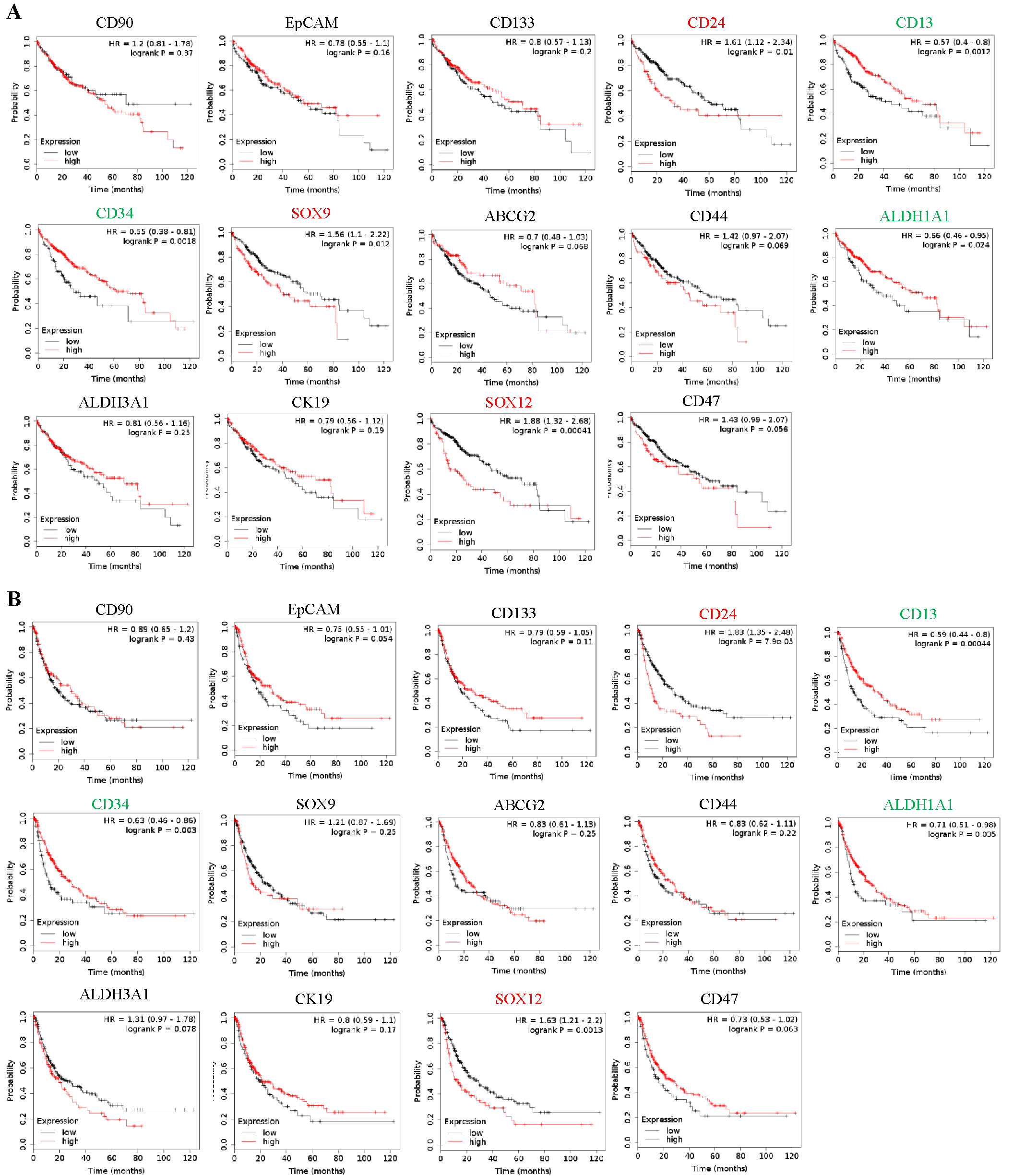

Supplement: Supplementary Figure 3 — Association of 14 HCSC markers expression levels and prognosis of HCC. (A) Correlation of HCSC markers high expression levels with OS of HCC, n=364, (B) correlation of HCSC markers high expression levels with PFS of HCC, n=370, red font means negative correlation, green font means positive correlation, black font means no correlation. [file Image_3.tif]

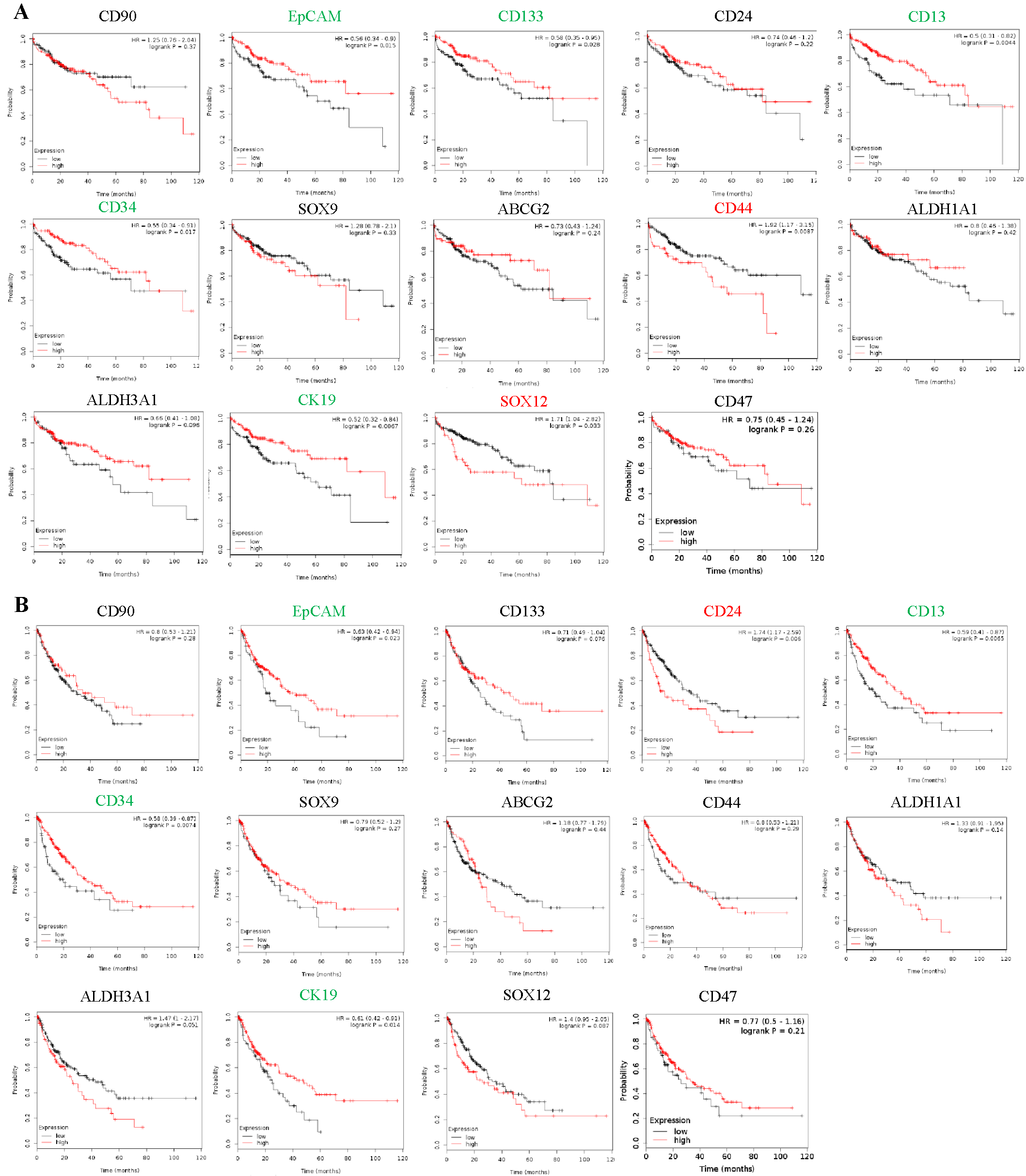

Supplement: Supplementary Figure 4 — Association of HCSC markers expression levels and prognosis of HCC in stage I and II. (A) Correlation of HCSC markers high expression levels with OS of HCC in stage I and II, n=253, (B) correlation of HCSC markers high expression levels with PFS of HCC in stage I and II, n=256, red font means negative correlation, green font means positive correlation, black font means no correlation. [file Image_4.tif]

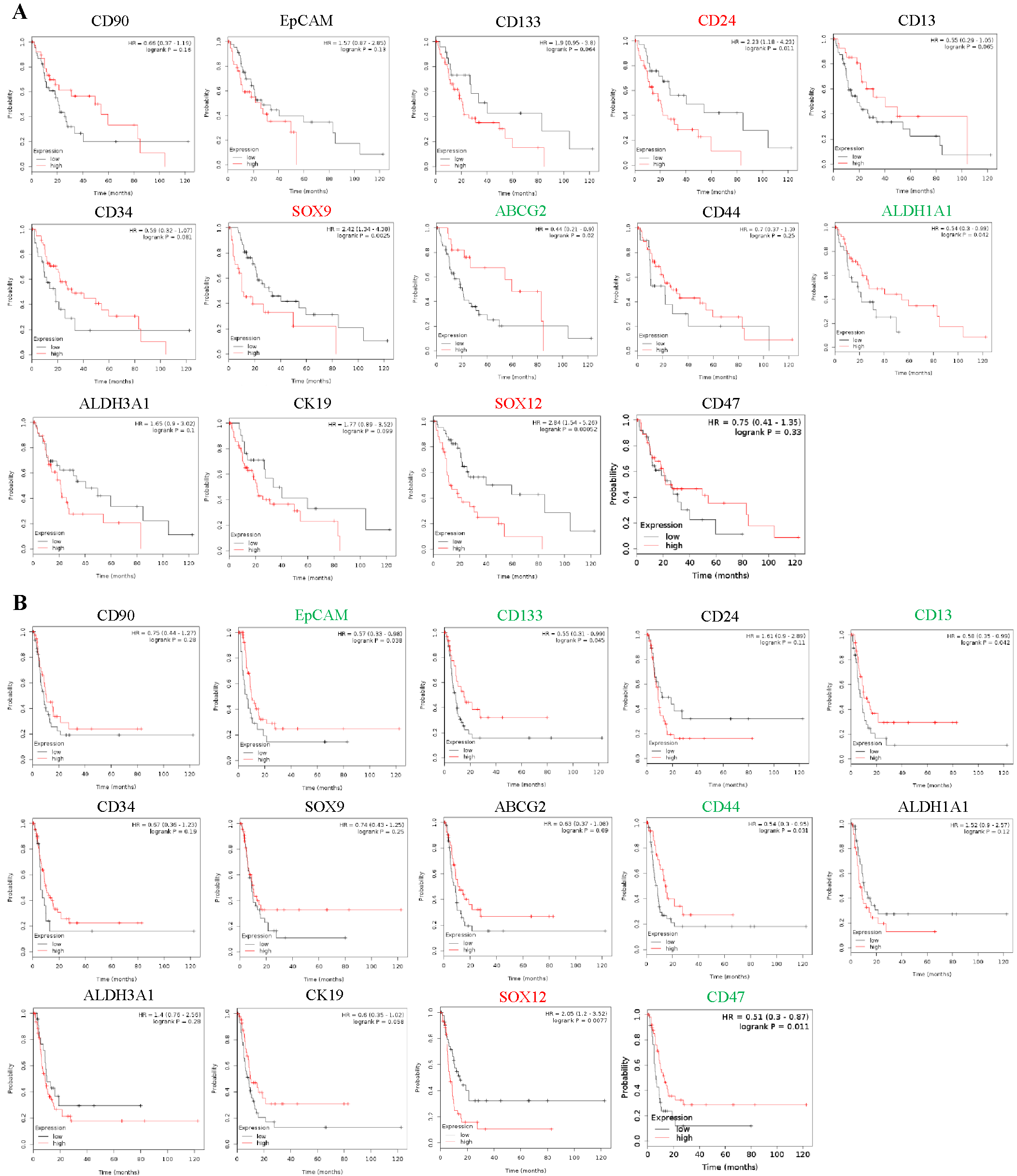

Supplement: Supplementary Figure 5 — Association of HCSC markers expression levels and prognosis of HCC in stage III and IV. (A) Correlation of HCSC markers high expression levels with OS of HCC in stage III and IV, n=87, (B) correlation of HCSC markers high expression levels with PFS of HCC in stage III and IV, n=90, red font means negative correlation, green font means positive correlation, black font means no correlation. [file Image_5.tif]

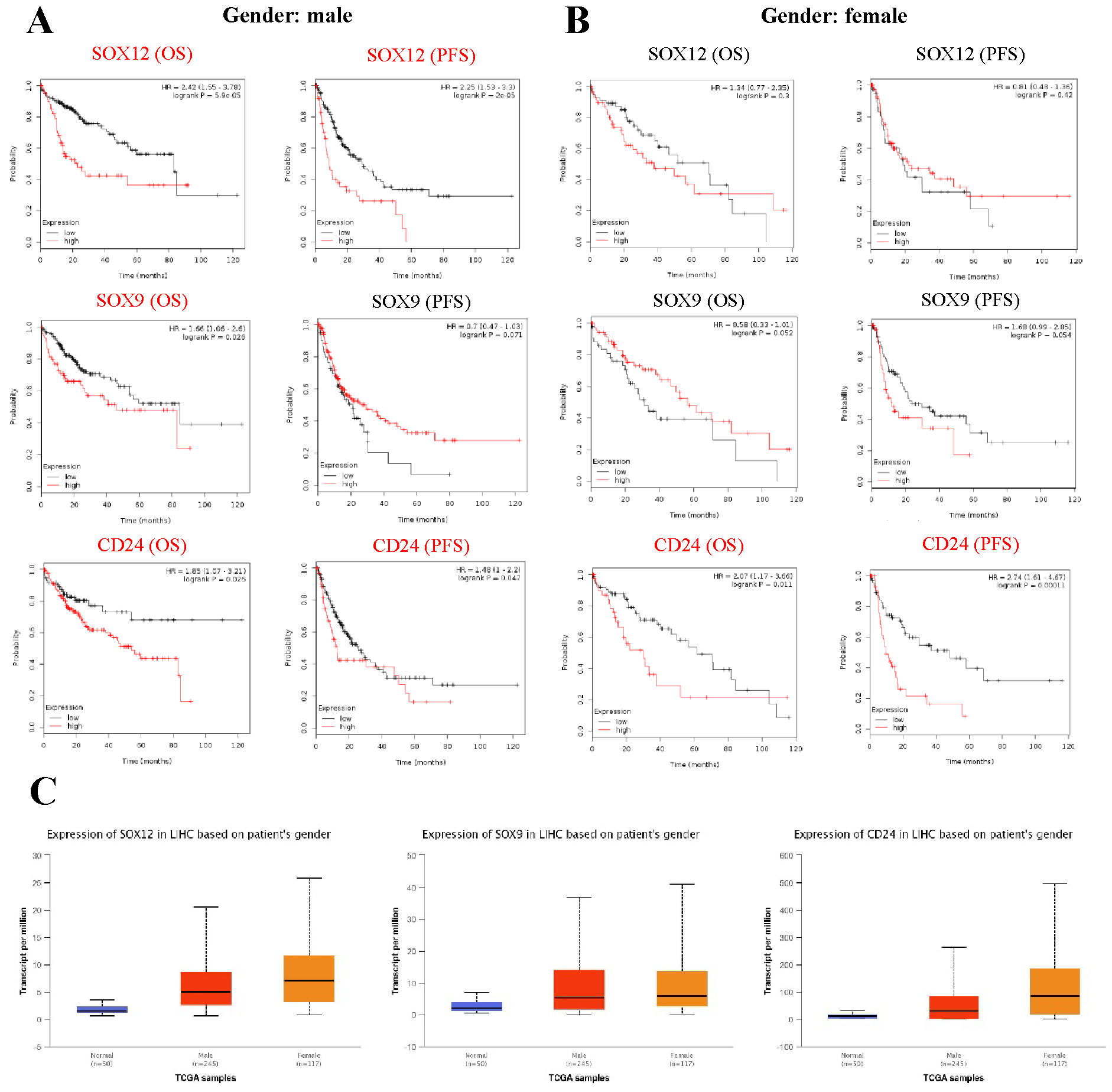

Supplement: Supplementary Figure 6 — Association of HCSC markers expression levels with prognosis of HCC with different gender. (A) Correlation of HCSC markers high expression levels with OS (n=246) and PFS (n=246) of male patient with HCC, (B) correlation of HCSC markers high expression levels with OS (n=118) and PFS (n=120) of female patient with HCC, red font means negative correlation, black font means no correlation. (C) Expression of HCSC markers in LIHC based on patient's gender. The median TPM and P value list in Table 2 . [file Image_6.tif]

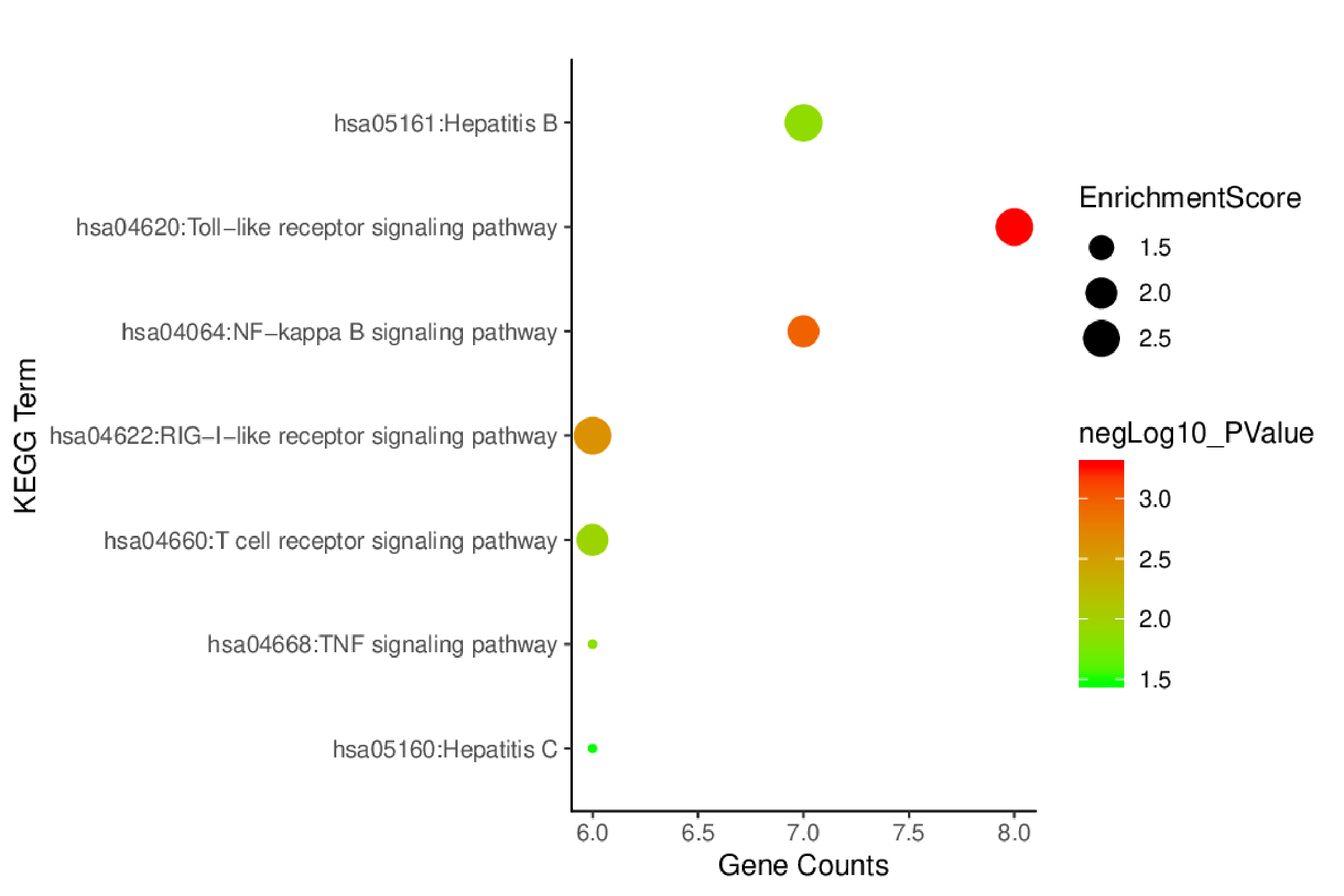

Supplement: Supplementary Figure 7 — KEGG enrichment plot of KEGG pathway by functional annotation clustering. [file Image_7.tif]
